# Computational Insights into the Antioxidant Activity of Luteolin: Density Functional Theory Analysis and Docking in Cytochrome P450 17A1

**DOI:** 10.3390/ph18030410

**Published:** 2025-03-14

**Authors:** Antônio Sérgio Nakao de Aguiar, Lucas Barbosa Ribeiro de Carvalho, Clayson Moura Gomes, Murillo Moraes Castro, Frederico Severino Martins, Leonardo Luiz Borges

**Affiliations:** 1 Laboratory of New Materials, Evangelical University of Goiás, Anápolis 75083-51, GO, Brazil; toninho.quimica@gmail.com; 2 Theoretical and Structural Chemistry Group of Anápolis, State University of Goiás, Anápolis 75132-903, GO, Brazil; 3 School of Medical and Life Sciences, Pontifical Catholic University of Goiás, Goiânia 74605-010, GO, Brazil; lubarica@gmail.com (L.B.R.d.C.); claysonmoura@yahoo.com.br (C.M.G.); drmurillomoraescastro@gmail.com (M.M.C.); 4 Department of Clinical and Toxicological Analysis, Faculty of Pharmaceutical Sciences, University of São Paulo, Av. Prof. Lineu Prestes, 580, Bl. 13B, São Paulo 05508-000, SP, Brazil; fredseverinomartins@gmail.com

**Keywords:** flavonoids, prostate cancer, bioactivity, binding affinity

## Abstract

**Background:** Luteolin, a flavonoid with well-documented antioxidant properties, has garnered significant attention for its potential therapeutic effects. **Objectives**: This study aims to investigate the antioxidant properties of luteolin under the influence of solvents, utilizing computational techniques to elucidate its interactions and its potential role as a modulator of enzymatic activities, particularly with Cytochrome 17A1. **Methods**: Density Functional Theory (DFT) calculations were employed to determine luteolin’s electronic and structural characteristics. Key aspects analyzed included electron density distribution and the energies of the frontier molecular orbitals (HOMO and LUMO). Free radical scavenging mechanisms were explored by comparing the dissociation enthalpy of the O–H bond in the absence and presence of water molecules. Additionally, molecular docking simulations were performed to assess the interactions of luteolin with Cytochrome 17A1, identifying preferred binding sites and interaction energies. **Results**: The findings indicate that luteolin possesses distinct structural and electronic features that contribute to its effectiveness in protecting against oxidative stress. However, hydrogen bonding interactions with water molecules were found to influence the dissociation enthalpy of the O–H bond. Docking simulations revealed significant interaction profiles between luteolin and Cytochrome 17A1, suggesting its potential role as a modulator of this protein. **Conclusions**: This study underscores the therapeutic potential of luteolin and highlights the importance of computational techniques in predicting and understanding the molecular interactions of bioactive compounds with biological targets. The results provide valuable insights that may aid in developing new therapeutic strategies for diseases associated with oxidative stress.

## 1. Introduction

Antioxidant compounds are crucial in protecting cells from oxidative stress, which is a key factor in the pathogenesis of various diseases, including cancer, cardiovascular disorders, and neurodegenerative conditions. Luteolin, a flavonoid widely found in fruits, vegetables, and medicinal plants, has garnered significant attention due to its diverse pharmacological properties, particularly its potent antioxidant activity. Several studies have confirmed the antioxidant potential of luteolin through assays such as DPPH• radical scavenging, ABTS radical scavenging, the FRAP test, and ferric ion reduction [1,2]. Additionally, quantum mechanical studies further support this property, elucidating its free radical scavenging mechanisms [3,4,5]. By neutralizing reactive oxygen species (ROS), luteolin helps mitigate cellular damage, improving overall health maintenance and disease prevention [1,2].

Recent advances in computational chemistry have significantly enhanced our understanding of the mechanisms by which natural compounds, such as luteolin, exert their biological effects. Among the most widely used methods for studying the molecular properties of chemical compounds—such as electronic structure, stability, and reactivity—is Density Functional Theory (DFT) [6,7]. Through DFT calculations, valuable insights can be obtained regarding molecular interactions at the atomic level, aiding in elucidating the mechanisms underlying the antioxidant activity of luteolin and other bioactive compounds. Several DFT studies have successfully described the free radical scavenging mechanisms of flavonoids [8,9,10] and other natural compounds [11].

Additionally, molecular docking studies have emerged as essential techniques for predicting how small molecules interact with target proteins. Cytochrome P450 enzymes, including Cytochrome 17A1, are crucial players in drug metabolism and the biotransformation of xenobiotics. Understanding the binding interactions between luteolin and Cytochrome 17A1 can shed light on its potential role as a modulator of enzymatic activity and a contributor to the antioxidant defense mechanisms in biological systems [12].

Several studies employ theoretical calculations to determine the antioxidant activity of flavonoids, considering the implicit presence of solvents, such as water [8,9,13]. However, an important question arises: How does the explicit presence of water molecules influence the antioxidant activity of these flavonoids? The present study investigates this effect using DFT calculations to address this question. Furthermore, we aim to provide deeper insights into its antioxidant mechanism by analyzing the electronic structure of luteolin and evaluating its binding affinity and interaction modes with Cytochrome 17A1. By integrating computational approaches, this study seeks to enhance our understanding of the structural and functional properties contributing to luteolin’s bioactivity, ultimately guiding the development of novel antioxidant therapies.

## 2. Results and Discussion

### 2.1. Molecular Structure Analysis

**Figure 1:**
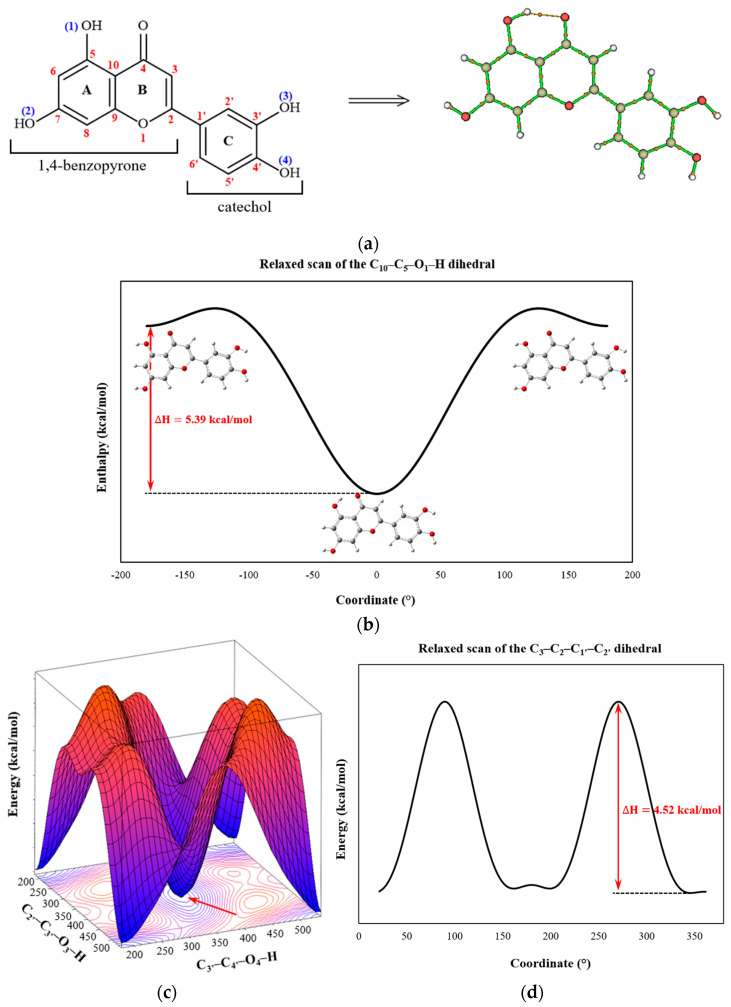
Geometric analysis of the luteolin molecule. (**a**) The structural formula of luteolin highlights the bond path (BP) between the 1-OH group and the 4-oxo function, indicating hydrogen bond formation. (**b**) Relaxed scan plot illustrating enthalpy variations as a function of 1-OH group rotation, where the red arrow marks the lowest energy point for the simultaneous rotations of the 3-OH and 4-OH groups. (**c**) Three-dimensional relaxed scan plot, where the red arrow marks the lowest energy point for the simultaneous rotations of the 3-OH and 4-OH groups. (**d**) Relaxed scan plot depicting the lowest energy state associated with the rotation of the C_2_–C_1′_ bond.

The luteolin molecule comprises a 1,4-benzopyrone ring and a catechol group (C) [14]. The 1,4-benzopyrone is formed by a benzene ring (A) fused to a pyrone ring (B), with the carbonyl group positioned at carbon 4. Two OH groups are bonded at positions 5 and 7 on ring A, while two additional OH groups are bonded at positions 3′ and 4′ on ring C (Figure 1a); the O–H bond lengths average 0.97 Å. According to optimization calculations, an interaction occurs between the 1-OH group and the 4-oxo function of the 1,4-pyrone moiety, contributing to the ground state stability of the molecule. The enthalpy difference betwdeen the bound and unbound conformations is 5.39 kcal/mol (Figure 1b). This interaction increases the bond length from 0.9669 Å (unbound) to 0.9843 Å (bound), representing a relative increase of approximately 1.8%.

Topological analyses using the QTAIMs indicated the presence of a bond path (BP) in the internuclear region between the H and O atoms responsible for forming the O_1_–H⋯O=C interaction, with an internuclear distance of 1.739 Å. According to the topological parameters on the bond critical point (BCP)—$\rho \left(r\right)=0.0425$; $\nabla^{2}\rho =0.1373$; h(r)=−0.0034; and |υ|/G=1.1—this is a moderate-intensity H-bond [15,16,17], characterized by partial covalency and an estimated binding energy of −8.7 kcal/mol, based on the following expression [18]:

(1) BE≈−223.08ρ(r)+0.7423,

where ρ(r) is the electron density. Furthermore, the dihedral angles of the –OH groups in the catechol moiety, C_2′_–C_3′_–O_3_–H and C_3′_–C_4′_–O_4_–H, are −1.35° and −1.88°, respectively, in the ground state (Figure 1c). Molecular topology analyses revealed no bond path (BP), indicating interaction between these two groups (Figure 1a).

The results also suggested that the C_3_–C_2_–C_1′_–C_2′_ dihedral angle is approximately 13.70°, not changing significantly in the presence of solvents (except in the case of benzene, where the molecule achieves its maximum planarity). This finding indicates that the catechol ring is slightly out of the plane of the 1,4-benzopyrone ring, a common characteristic of many flavonoid molecules. Conversely, the most energetic conformation occurs when the rings are nearly perpendicular, with an enthalpy difference of approximately 4.52 kcal/mol (Figure 1d). This slight torsion in the molecule significantly influences the antioxidant activity of flavonoids, as the planarity of the molecular system facilitates conjugation, delocalization, and stabilization of the phenoxyl radical formed during free radical scavenging [19]. Nevertheless, the low enthalpy value permits ring rotations in solution or physiological media.

**Figure 2:**
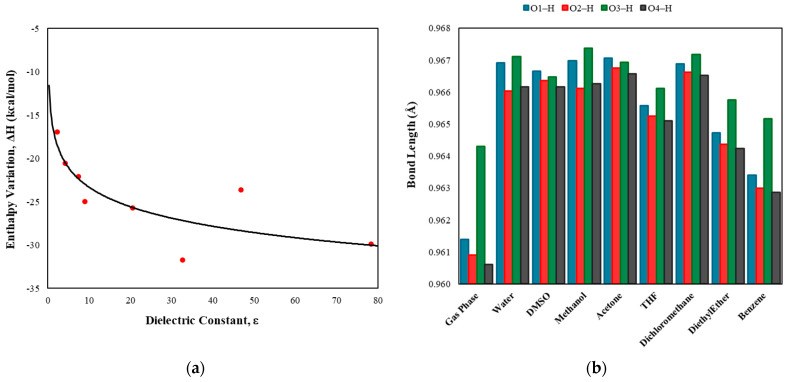
Graphical representation of solvent effects on luteolin. (**a**) Variation in the relative enthalpy (ΔH) as a function of solvent dielectric constant, with values obtained using Equation (2). (**b**) Bond length variations in the O*_x_*–H groups in luteolin across different solvents.

The calculation of the relative enthalpy in different environments was performed using the following equation:

(2) $$∆H=H_{solution}-H_{vacuum},$$

where $H_{solution}$ is the enthalpy of luteolin in various solvents and $H_{vacuum}$ is its enthalpy in a vacuum. The results showed that the flavone molecule stabilizes as the dielectric constant of the solvent increases (Figure 2). This stabilization is attributed to strong interactions, such as hydrogen bonds, with polar solvents, which contribute to the stabilization of the molecular system. Additionally, these interactions lead to an increase in the lengths of the O*_x_*–H bonds. For example, in the presence of the most nonpolar solvent in this study (benzene), the bond lengths are shorter than those in polar solvents but remain slightly longer than those observed in a vacuum. This indicates that the OH groups still engage in some level of interaction with nonpolar solvents.

### 2.2. Electronic Structure Analysis

**Figure 3:**
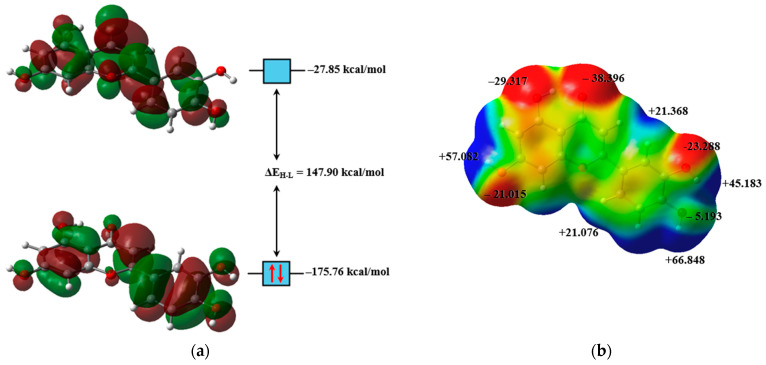
Visualization of the electronic properties of luteolin. (**a**) Isosurfaces of the frontier molecular orbitals, HOMO and LUMO (isovalue = 0.02 a.u.), illustrating electron density distribution and molecular reactivity. (**b**) A Molecular Electrostatic Potential map at ρ(r) = 4.0 × 10⁻⁴ electrons/Bohr^3^ contour of the total SCF electron density, highlighting regions of electrophilic and nucleophilic character. Calculations were performed at the M06-2X/6-311++G(d,p) level of theory.

**Figure 4:**
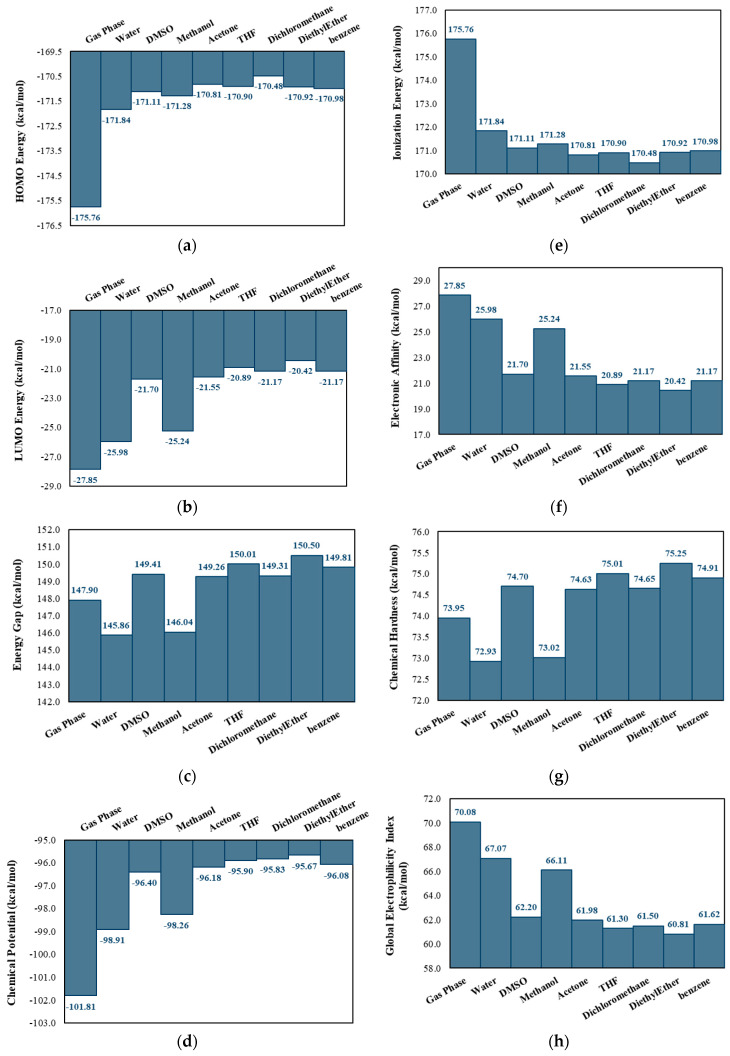
Bar graphs illustrating the variations in (**a**) HOMO energy, (**b**) LUMO energy, (**c**) energy gap, (**d**) chemical potential, (**e**) ionization energy, (**f**) electron affinity, (**g**) chemical hardness, and (**h**) global electrophilicity index in the gas phase and as a function of the dielectric constants of the solvents.

The isosurfaces of the frontier molecular orbitals of luteolin are shown in Figure 3a, and both orbitals are classified as π orbitals. Calculations suggest that luteolin has a reduced capacity to stabilize the HOMO and LUMO in nonpolar solvents, resulting in higher orbital energies (Figure 4a,b). This instability decreases the ionization energy (Figure 4e), facilitating electron removal and enhancing its effectiveness as a reducing agent. Conversely, in polar solvents, the interactions between the compound and the solvent, primarily through H-bonds, stabilize the energies of these orbitals. H-bonds are attractive interactions between a hydrogen atom bonded to a more electronegative element (X–H) and another atom or group of atoms, either within the same molecule or in a different molecule, provided there is evidence of bond formation [20]. This stabilization increases the electron affinity of luteolin, enhancing its oxidizing character (Figure 4f). Additionally, the system’s stabilization in polar solvents can facilitate electronic transitions [21,22], thereby increasing the molecule’s reactivity in specific reactions [23].

Nevertheless, an anomaly was observed among the three most polar solvents (water, DMSO, and methanol), where the orbital energies do not precisely follow the expected trend. This anomaly can be explained by the nature of the intermolecular interactions established with luteolin. First, it is essential to note that water and methanol molecules interact with the –OH groups of luteolin through two interactions: O–H⋯O*_solvent_* and O⋯H–O*_solvent_*, while DMSO interacts solely through O–H⋯O*_d_* interactions. The symbols (–) and (⋯) represent the chemical bond and the interaction, respectively. The absence of O⋯H–O*_d_* interactions limits the additional stabilization of the luteolin–DMSO system.

All luteolin–solvent interactions were estimated using the MEP surface of luteolin (Figure 3b), where the electrophilic regions of the molecule are represented in red and the nucleophilic areas in dark blue. The MEP map indicates that luteolin’s molecular polarity index (MPI) is 12.68 kcal/mol, with its polar area comprising 43.01% of the molecular surface [24]. For comparison, the MPI values calculated for water and benzene were 23.09 and 8.15 kcal/mol, respectively, suggesting that the luteolin molecule exhibits medium polarity.

**Figure 5:**
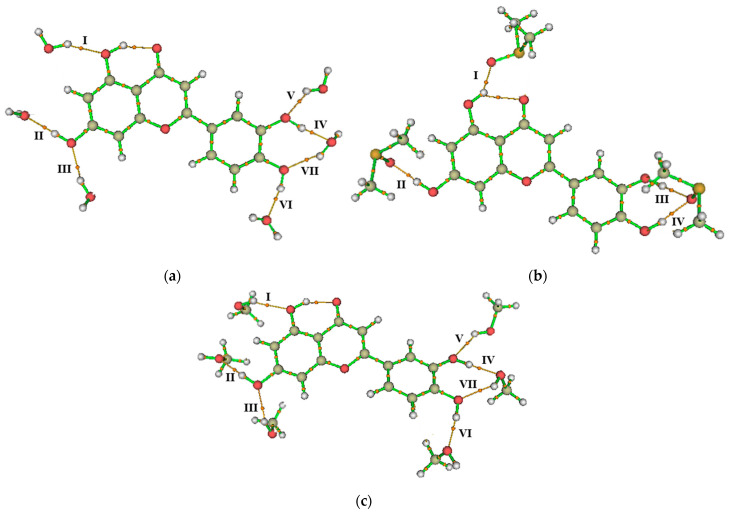
Molecular graphs depicting the bond paths formed by the intermolecular interactions between the OH groups of luteolin and molecules of (**a**) water, (**b**) DMSO, and (**c**) methanol. Orange lines represent the bond paths, while orange spheres indicate critical binding points, while orange spheres represent the bond critical points (indicated by the Roman numerals).

After optimizing the luteolin–solvent complexes, it was observed that each OH group interacts directly with up to two water or methanol molecules, except for the 1-OH group, which interacts with only one solvent molecule. As previously mentioned, this occurs due to its interaction with the 4-oxo function. In the case of DMSO, only one solvent molecule interacts directly with each OH group. Additionally, the oxygen atom of DMSO shares a bifurcated interaction with the 3-OH and 4-OH groups, contributing to the additional stabilization of these groups through a single solvent molecule. The remaining solvent molecules interact in the medium, and weaker interactions were observed in other regions of the flavone. Due to their low relevance, these interactions were not discussed in this work. Figure 5 presents the interaction diagram of the studied complexes.

The topological parameters of the QTAIMs are presented in Table 1. The internuclear regions of the O–H⋯O*_solvent_* interactions exhibit slightly higher charge density than the O⋯H–O solvent interactions, significantly stabilizing the interacting systems. The 3-OH group interacts with the solvent molecule, resulting in binding energies (BE) of −14.00 kcal/mol for water, −13.85 kcal/mol for DMSO, and −14.13 kcal/mol for methanol. Additionally, the interaction distances are similar, with the interaction of luteolin with water showing a slightly larger distance (~1.77 Å). The electron densities at BCP and the bonding distances between luteolin and water molecules exhibit values comparable to those observed for other previously studied flavonoids [25,26,27].

NBO analyses revealed that the bonding orbital η(O*_solvent_*) hyperconjugated with the antibonding orbital $\sigma^{*}$(O*_x_*–H) of the OH group of luteolin, η(O*_solvent_*) → $\sigma^{*}$(O*_x_*–H) [18,22]. Table 2 presents the results of the NBO analysis. The stabilizing energies associated with these interactions, $E_{i\to j^{*}}^{\left(2\right)}$, were high, indicating a tendency toward a partially covalent character (Table 1). The data showed that the interactions of the 3-OH group with water and methanol presented the highest $E_{i\to j^{*}}^{\left(2\right)}$ values (21.00 and 18.63 kcal/mol, respectively), indicating that the O_3_H⋯O*_solvent_* interactions are the most stable. In the case of DMSO, the greatest hyperconjugation occurs in the interaction with the 2-OH group, with a $E_{i\to j^{*}}^{\left(2\right)}$ value of 20.57 kcal/mol. The bifurcated interaction of DMSO with the 3-OH and 4-OH groups contributes to more localized stabilization, resulting in BE values of −13.85 kcal/mol for 3-OH and −14.22 kcal/mol for 4-OH. The stabilizing energies for these interactions are also high, with values of 12.72 and 14.74 kcal/mol, respectively. Although these interactions are less diverse than those observed in water and methanol, they reinforce DMSO’s stabilizing role in specific luteolin regions.

The distance of the O_3_⋯H–O*_w_* (*w* = water) interaction is ~2.16 Å, with a BE of −7.95 kcal/mol. This interaction is stabilized by the hyperconjugation $\eta_{2}$(O_3_) → $\sigma^{*}$(O*_w_*–H), with a $E_{i\to j^{*}}^{\left(2\right)}$ value of 2.56 kcal/mol. For O_3_⋯H–O*_m_* (*m* = methanol), the distance is slightly shorter (~2.00 Å), with a BE of −8.03 kcal/mol, stabilized by the hyperconjugation $\eta_{2}$(O_3_) → $\sigma^{*}$(O*_m_*–H), with a $E_{i\to j^{*}}^{\left(2\right)}$ value of 2.07 kcal/mol. However, due to its shorter interaction and higher electron density at the BCP, methanol stabilizes the O_3_H group more efficiently.

These strong interactions with water may have subtle implications for the antioxidant activity of luteolin, as the stabilization promoted by the solvent could modulate the reactivity of the phenolic groups, potentially affecting the hydrogen donation mechanisms.

The stabilization of both the HOMO and the LUMO orbitals, as well as the other descriptors discussed in protic polar solvents, does not imply lower reactivity. Instead, it favors chemical interactions by reducing the $∆E_{H-L}$ (Figure 4c) and η (Figure 4g). In water and methanol, H-bonds more effectively stabilize the electronic states of luteolin. However, despite strong interactions in DMSO, the absence of additional H-bonds renders this solvent less effective in stabilizing the electronic states, resulting in the distinct behavior observed. The lack of these additional interactions in DMSO leads to a larger $∆E_{H-L}$ and increased η, which are characteristics associated with greater stability and lower reactivity. Therefore, the electronic behavior of luteolin in different solvents underscores how intermolecular interactions modulate its reactive properties and its ability to act as either a reductant or an oxidant, depending on the polarity and nature of the solvent.

### 2.3. Antioxidant Potential Analysis

**Figure 6:**
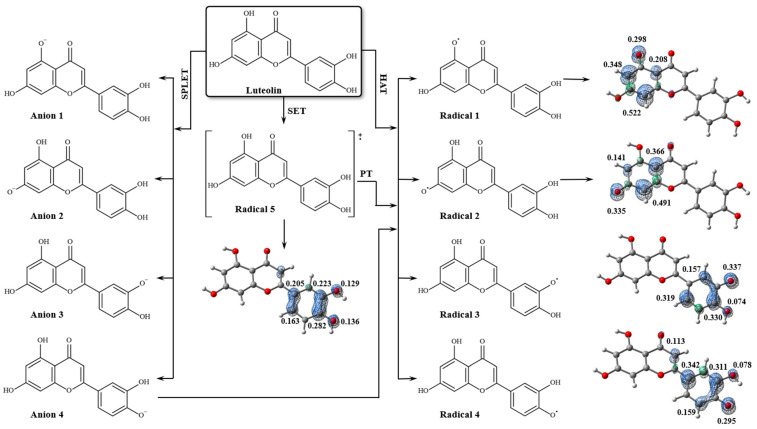
Chemical species formed from luteolin in each of the free radical scavenging mechanisms and graphs of electron spin densities for the unpaired electron in each of the species (ρ(r) = 0.006 a.u.).

The luteolin molecule can form four distinct A∙ radicals during the free radical scavenging process, as shown in Figure 6. The results of theoretical calculations for the free radical scavenging mechanisms are presented in Table 3, indicating that the HAT mechanism is preferred for luteolin. The O_3_–H bond exhibits the lowest BDE value, requiring a dissociation energy of 80.66 kcal/mol. Electron spin density analysis revealed that the unpaired electron is resonance-stabilized at the catechol moiety in Radical 3. Previous studies have demonstrated that the high antioxidant activity of luteolin is primarily attributed to the catechol moiety, combined with the unsaturation at the 2,3-position and the 4-oxo function of the 1,4-pyrone moiety [28]. This structural arrangement facilitates efficient electronic conjugation between rings A and C, promoting electronic delocalization through the conjugated π system. The 3-OH group is particularly significant due to its position within the conjugated system, enabling stabilization of the generated phenoxyl radical, reducing its energy, and enhancing the antioxidant efficiency of luteolin.

The O_4_–H bond also contributes to the antioxidant activity of luteolin. However, the dissociation enthalpy of the O_4_–H bond is slightly higher (~3.6%) because the electron density of the O_4_ atom is conjugated to the π system of the pyrone ring (ring B). The 4-oxo function, however, influences the delocalization of electron density, slightly reducing the hydrogen donation capacity. Calculations revealed that the unpaired electron in Radical 4 is stabilized by resonance within the C ring.

Although the 1-OH and 2-OH groups may also contribute to the antioxidant activity of luteolin, their lower capacity to stabilize the unpaired electron increases the BDE values by 9.2% and 14.2%, respectively, thereby reducing their chemical reactivity. The position of these groups within the compound’s structure limits the electronic delocalization of the oxygen atoms in Radicals 1 and 2 (Figure 6), due to their restricted interaction with the π system. Calculations revealed that the electron spin density is not uniformly distributed across the benzopyrone ring, unlike the catechol group.

Commercial antioxidant compounds widely used as preservatives in food, cosmetics, pharmaceuticals, and biodiesel—including butylhydroxyanisole (BHA), butylhydroxytoluene (BHT), gallic acid (GA), propyl gallate (PG), pyrogallol (PY), and tert-butylhydroquinone (TBHQ)—exhibit BDE values ranging from 76 to 85 kcal/mol (Table 3). When comparing these values with the phenolic groups of luteolin, it is observed that the 3-OH and 4-OH groups fall within the same range of antioxidant activity as these additives. In contrast, the 1-OH and 2-OH groups confirm previous discussions, indicating their lower contribution to the molecule’s overall antioxidant activity.

The results showed that H-bonds formed with water molecules slightly impact the dissociation energies of the O*_x_*–H bonds in luteolin, making some interactions more cohesive while weakening others (Table 3). The average BDE values did not change significantly, showing an overall increase of only 0.75%. However, data dispersion increased by 7.06% in interactions with the solvent, with the standard deviation rising from 5.04 kcal/mol (without water) to 5.40 kcal/mol (with water). The O_3_–H bond exhibited the most significant increase in BDE (~2.60%), whereas the O_1_–H group was the least affected by the attractive effects of H-bond with water, showing an increase of only ~0.69% in BDE. The low sensitivity of this group to the solvent’s presence can be attributed to its intramolecular interaction with the 4-oxo function, which partially stabilizes the bond. Conversely, the O_4_–H bond became more accessible for interactions with free radicals, leading to decreased cohesion energy (~1.80%). This reduction in BDE suggests that the 4-OH group plays a more significant role in the antioxidant mechanisms of luteolin, potentially enhancing its ability to neutralize reactive species.

The calculations indicated that two water molecules interact with the O_1_**∙**, O_2_**∙**, and O_4_**∙** atoms, while only one interacts directly with O_3_**∙**. The topological parameters showed that the charge density is higher in the [O*_x_***∙**]⋯H–O*_w_* interactions than in the O*_x_*⋯H–O*_w_* interactions, confirming their H-bond nature. By comparing the ρ and BE values in the AH/H_2_O and A**∙**/H_2_O systems, an increase in intermolecular attractive forces was observed in the A**∙** radicals, suggesting greater stabilization of these systems in aqueous media.

**Figure 7:**
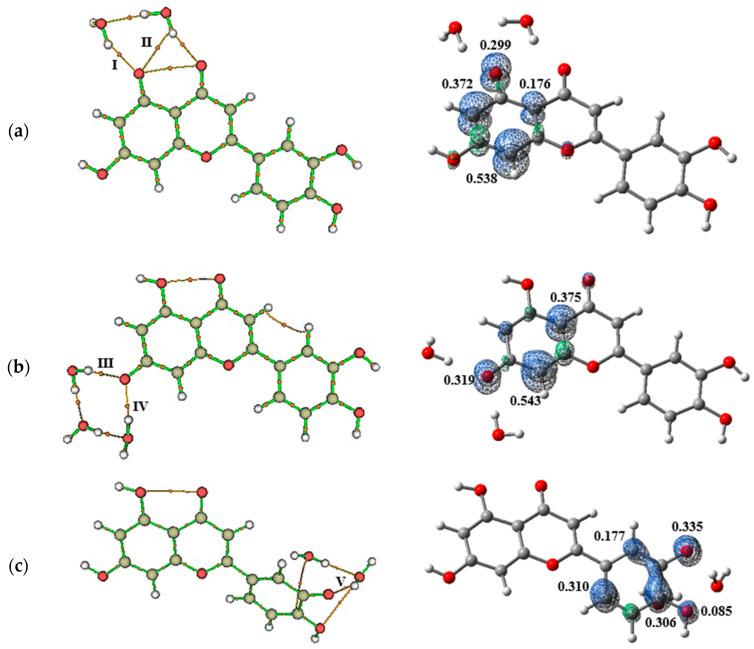

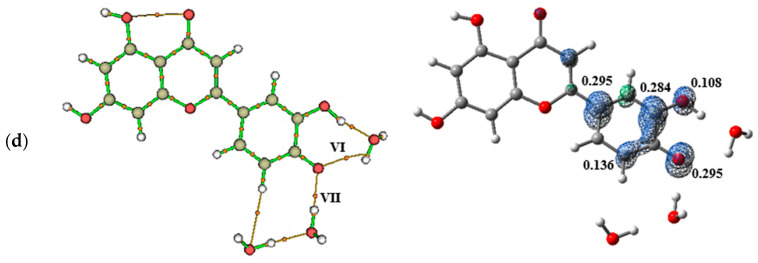
At left, bond paths formed between (**a**) O_1_**∙**, (**b**) O_2_**∙**, and (**c**) O_3_**∙** and (**d**) O_4_**∙** radicals and water. At right, spin density distribution revealed that interactions with water molecules did not alter the stabilities of the radicals formed during the free radical scavenging process. Orange lines represent the bond paths, while orange spheres represent the bond critical points (indicated by the Roman numerals).

Additionally, it was found that a water molecule shares an interaction between O_1_**∙** and the carbonyl group (C=O), leading to electronic depletion in the bond critical point (BCP) region of the [O_1_**∙**]⋯H–O*_w_* (II) interaction, characterizing a van der Waals interaction (Figure 7). NBO analysis revealed that, unlike the interactions of the AH molecule with water, the stabilizing energy of these interactions in the radicals is very low, indicating that solvation contributes minimally to the stabilization of A radicals (Table 2). The spin density distribution revealed that interactions with water molecules did not alter the stabilities of the radicals formed during the free radical scavenging process (Figure 7).

These findings align with previous studies discussing the role of non-covalent interactions, particularly H-bonds, in modulating the reactivity of phenolic antioxidants [29,30,31]. Whether intramolecular or involving the surrounding environment, H-bonds significantly influence antioxidant activity by altering the molecule’s ability to donate or accept electrons when reacting with radical species. In luteolin, the observed changes in BDE can be explained by two main effects: (i) H-bonds involving the reactive −OH groups can reduce antioxidant efficiency if the −OH acts as an H-bond donor, whereas the opposite effect generally occurs when it serves as an H-bond acceptor; (ii) remote intra- or intermolecular H-bonds, such as those involving the 4-oxo function, can either enhance or diminish antioxidant reactivity, depending on whether the stabilization provided by the H-bond increases or decreases along the reaction coordinate, from the reactants to the transition state.

### 2.4. Molecular Docking and Pharmacophore Modeling

**Figure 8:**
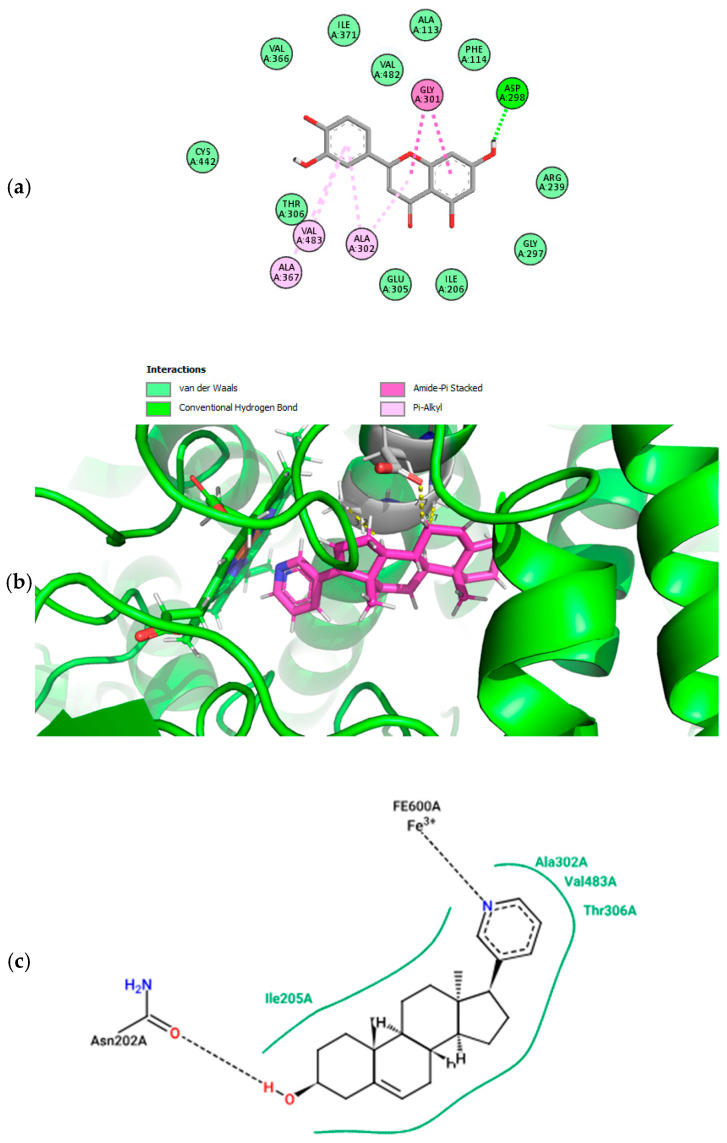
Molecular docking analysis of luteolin and abiraterone in the active site of Cytochrome P450 CYP17A1. This figure provides insights into the interaction patterns and binding conformations of luteolin compared to a known inhibitor. (**a**) Two-dimensional interaction diagram of luteolin bound to Cytochrome P450 CYP17A1 in conformation 1, generated using Discovery Studio 3.5 Visualizer. (**b**) Luteolin docked in the ligand-binding site of Human Cytochrome P450 CYP17A1 in conformation 1, visualized with Pymol 3.1.3. Distance values are given in Å. (**c**) Two-dimensional interaction map of abiraterone co-crystallized with the active site of Cytochrome P450 CYP17A1, generated using PoseView (https://proteins.plus/ accessed on 18 January 2025).

Based on evidence from Sakurai and colleagues (2014) regarding the potential interaction of luteolin with prostate cancer targets such as CYP17A1, this target was selected for molecular docking and subsequent pharmacophore analysis [32]. Redocking of the co-crystallized ligand (abiraterone) with CYP17A1 was successfully performed [33]. This model was also employed for luteolin docking. All RMSD values were less than 0.6 A. In silico predictions have shown that luteolin fits well in the ligand binding site of Human Cytochrome P450 CYP17A1 (Figure 8a). The residues GLY301, ALA302, and VAL483 contact the luteolin and may contribute to orienting ligands within the binding site by hydrophobic interactions. The residue ASP298 seems to play an essential role in stabilizing the ligand in the binding site via hydrogen bond (Figure 8). The fitscore values for the best poses of abiraterone (co-crystallized ligand) and luteolin were 92.77 and 63.11, respectively.

The natural product luteolin exhibits a broad spectrum of bioactivity. The evidence that luteolin acts as an agent capable of preventing prostate cancer raises the hypothesis that this molecule interacts with some targets of this disease, particularly the potential inhibition of CYP450 17A1 [34,35]. The results suggested that luteolin’s chemical structure can interact with CYP450 17A1, making it a possible candidate to influence and prevent prostate cancer [33,36].

Moreover, the antioxidant effect of luteolin is significant in the context of cancer prevention. Antioxidants are crucial in neutralizing reactive oxygen species (ROS) that can cause cellular damage and contribute to carcinogenesis. By reducing oxidative stress, luteolin may protect cells from damage and inhibit pathways that lead to cancer progression. Therefore, luteolin represents an intriguing starting point for future investigations aimed at developing new therapeutic strategies for the treatment and prevention of prostate cancer.

**Figure 9:**
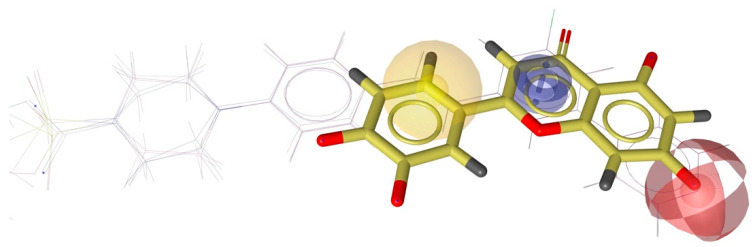
Three-dimensional model of shared featured pharmacophore between luteolin and the Cytochrome P450 CYP17A1 ligands. The pharmacophore features are color-coded for hydrogen-bond acceptors (red), hydrophobic (yellow), and aromatic rings (blue). The five compounds employed in this analysis were BDBM330383, BDBM330386, BDBM330388, BDBM330389, and BDBM330390 (these are the codes of the compounds found on the website https://www.bindingdb.org/rwd/bind/index.jsp accessed on 9 February 2025) [37].

**Figure 10:**
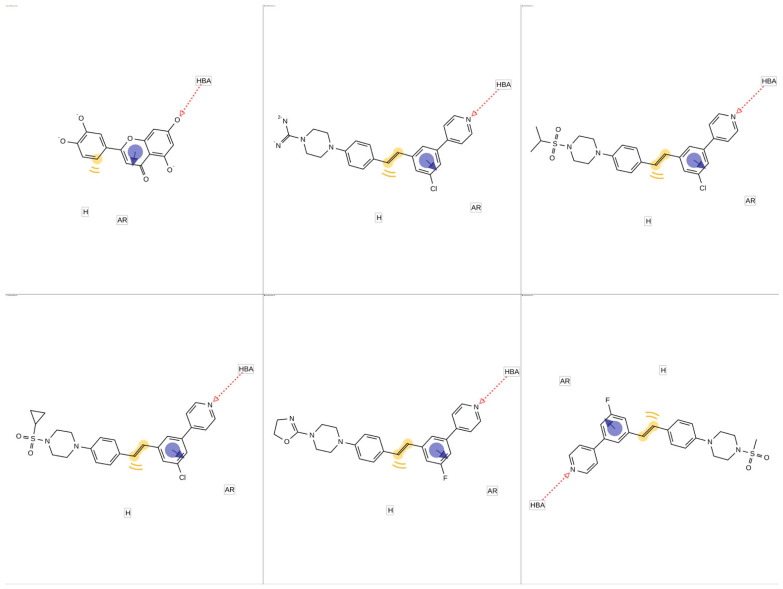
Individual pharmacophores of luteolin and five Cytochrome P450 CYP17A1 ligands with the lowest IC_50_. Hydrogen-bond acceptor (HBA), hydrophobic (H), and aromatic ring (AR).

Pharmacophore analysis is vital for gaining insight into the molecular characteristics of Cytochrome P450 17A1 ligands, which is critical for effective drug design. Each pharmacophore model, created using LigandScout software, emphasizes key features such as hydrogen bond acceptors (HBAs), hydrogen-bond donors (HBDs), aromatic rings (ARs), and hydrophobic (HyPho) regions. These features are visually represented in Figure 9 and Figure 10 as follows: the pharmacophore features are color-coded for hydrogen-bond acceptors (red), hydrophobic (yellow), and aromatic rings (blue).

Three features were shared between the luteolin molecule and the five ligands, suggesting the potential for interactions between this structure and the Cytochrome P450 17A1 (Figure 9 and Figure 10).

The results of the pharmacophore modeling help to corroborate the data from the molecular docking, particularly regarding the role of the hydrogen bond between the luteolin molecule and ASP298, as well as the role of aromatic and hydrophobic interactions with the ALA302 residue. If we observe Figure 8c, the amino acids ALA302, VAL483, and THR306 interact with the pyridinic ring of abiraterone (the co-crystallized ligand). This was also confirmed for the docking of luteolin. The hydrogen bond of abiraterone occurs with the residue ASN202, whereas luteolin seems to happen with ASP298. The results suggest that a hydrogen donor group appears crucial in anchoring at the active site of the target CYP450 17A1.

## 3. Materials and Methods

### 3.1. Molecular Modeling

All theoretical calculations were performed using DFT [6,7], as implemented in the Gaussian 16 [38] software package. The luteolin molecule was constructed in GaussView 6.0 [39], and its geometry was optimized using the highly parameterized empirical exchange-correlation functional M06-2X [40], combined with the diffuse and polarized basis set 6-311++G(d,p). Studies have demonstrated that this functional reliably describes mid-range electronic correlation effects and non-covalent interactions and is one of the best-performing functionals for modeling the thermodynamics of chemical processes [41,42].

The reactivity of luteolin was studied through the energies of the frontier molecular orbitals, specifically the Highest Occupied Molecular Orbital (HOMO) and the Lowest Unoccupied Molecular Orbital (LUMO) [43]. From these, reactivity descriptors were obtained, including chemical hardness [44,45],

(3) $$\eta =\frac{1}{2}{\left(\frac{\partial^{2}E}{\partial N^{2}}\right)}_{\upsilon }=\frac{I-A}{2},$$

which measures the resistance to deformation of the electron cloud during chemical processes; chemical potential [45],

(4) $$\mu ={\left(\frac{\partial E}{\partial N}\right)}_{\upsilon }=-\frac{I+A}{2}=-\chi ,$$

which is related to charge transfer from a species with higher chemical potential ($\mu_{large}$) to one with lower chemical potential ($\mu_{small}$); and the global electrophilicity index [46],

(5) $$\omega =\frac{\mu^{2}}{2\eta },$$

which measures energy stabilization when the system acquires electronic charge from the environment. In Equations (3) and (4), E is the energy of the system, N is the number of particles, υ is the external potential, χ is the electronegativity, $I\approx -E_{HOMO}$ is the ionization potential, and $A\approx -E_{LUMO}$ is the electron affinity. The nucleophilic and electrophilic regions of the compound were evaluated using the Molecular Electrostatic Potential (MEP) map [47,48], where the electrostatic potential, V(r), at point r, is defined by the following:

(6) $$V(r)=\sum_{\alpha }\frac{Z_{\alpha }}{\left|r_{\alpha }-r\right|}-\int \frac{\rho \left(r^{\prime }\right)}{\left|r^{\prime }-r\right|}dr^{\prime },$$

where $Z_{\alpha }$ is the charge of nuclei α at point $r_{\alpha }$ and $\rho \left(r^{\prime }\right)$ is the charge density at the point $r^{\prime }$ [47].

Some researchers have reported that solvent molecules significantly affect the molecular properties of the solute and may influence the strength and antioxidant properties of compounds [49,50,51]. This is attributed to the solvent’s impact on the strength and nature of its interactions with the solute. In this context, the influence of solvents on the chemical reactivity of luteolin was calculated using implicit solvents, including water (ε = 78.3553), dimethyl sulfoxide (DMSO, ε = 46.826), methanol (ε = 32.613), acetone (ε = 20.493), tetrahydrofuran (THF, ε = 7.4257), dichloromethane (DCM, ε = 8.93), diethyl ether (ε = 4.2400), and benzene (ε = 2.2706). These calculations employed the continuum solvation model, SMD [45], based on a self-consistent reaction field treatment of bulk electrostatics.

Additionally, systems with the three most polar solvents (water, DMSO, and methanol) were constructed to examine how intermolecular interaction patterns directly influence luteolin’s reactivity. The molecular systems were constructed in such a way that solvent molecules were added one at a time to each OH group of luteolin and then subjected to optimization. The intermolecular interactions at the bond critical point (BCP) were analyzed employing the Quantum Theory of Atoms in Molecules (QTAIMs) [52,53], where the topological parameters were obtained by Multiwfn 3.3.8 suite [54]. The topological parameters collected were the electron density (ρ(r)), the Laplacian of electron density ($\nabla^{2}\rho$), the Lagrangian kinetic energy (G(r)), the potential electron density (v(r)), and the energy density (h(r)), which were used to describe the nature of the intramolecular H-bond interaction. The stability of these interactions was evaluated through Natural Bond Orbital (NBO) analysis [55,56], in which the donor and acceptor interaction energy was estimated using the following second-order perturbation formula:

(7) $$E_{i\to j^{*}}^{\left(2\right)}=-n_{\sigma }\frac{\left\langle \sigma_{i}|\hat{F}|\sigma_{j^{*}}\right\rangle }{\varepsilon_{j^{*}}-\varepsilon_{i}}=-n_{\sigma }\frac{F_{ij}^{2}}{\varepsilon_{j^{*}}-\varepsilon_{i}},$$

where $\left\langle \sigma_{i}|\hat{F}|\sigma_{j^{*}}\right\rangle$ (or $F_{ij}^{2}$) is the Fock matrix element between the i and j NBOs; $\varepsilon_{\sigma^{*}}$ is the energy of the antibonding orbital $\sigma^{*}$=; $\varepsilon_{\sigma }$ is the energy of the bonding orbital σ; and $n_{\sigma }$ is the population occupation of the σ donor orbital.

### 3.2. Antioxidant Potential Analysis

To determine the antioxidant potential of luteolin, thermodynamic descriptors were calculated with the molecule implicitly immersed in water. Subsequently, water molecules were added, and the systems were optimized to identify the ground state. Previous calculations showed that only two water molecules interact directly with each OH group of luteolin (except for the 1-OH group). Thermodynamic descriptors were recalculated and compared with those of the previous systems to evaluate the influence of direct water interactions on the antioxidant potential of the flavone. Additionally, electron spin density calculations were performed to assess the stability of the A∙ radicals formed during the free radical scavenging process.

**Figure 11:**
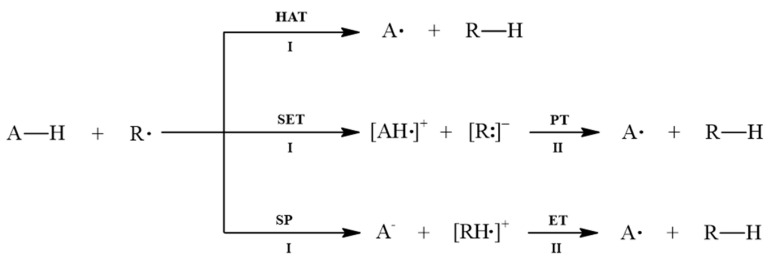
Diagram of free radical scavenging mechanisms.

There are several methods for determining the antioxidant activity of compounds [57]. Two main mechanisms can explain this property: hydrogen atom transfer (HAT) and proton transfer [58,59]. Hydrogen transfer processes can occur through homolytic or heterolytic scission of hydrogen atoms. The heterolytic pathway involves either a single electron transfer followed by proton transfer (SET-PT) or sequential proton loss followed by electron transfer (SPLET). The schematic representation of both mechanisms is shown in Figure 11.

In the HAT mechanism, the free radical R**⋅** abstracts a hydrogen atom from the antioxidant compound A–H, forming the new radical A**⋅** and the neutral compound R–H. The dissociation of the O–H bond is quantified by the bond dissociation enthalpy (BDE), as defined by the following equation:

(8) $$BDE=H_{A\cdot }+H_{H\cdot }-H_{AH}$$

where $H_{AH}$, $H_{A\cdot }$, and $H_{H\cdot }$ represent the enthalpies of the antioxidant, the radical A**⋅**, and the hydrogen atom, respectively. This thermodynamic descriptor has been widely used to evaluate the H atom abstraction capacity of antioxidants and ${BDE}_{min}$ identifies the H-donating group most susceptible to radical attacks.

SET-PT is another mechanism through which antioxidant compounds act. This mechanism occurs in two steps: in the first step, an electron is transferred from the antioxidant to the free radical, forming the radical cation [AH**⋅**]^+^ and the anion [R∶]^−^. In the second step, a proton is transferred from [AH·]^+^ to [R∶]^−^, forming A**⋅** and R–H. The electron transfer is characterized by the ionization potential as follows,

(9) $$IP=H_{{\left[AH\cdot \right]}^{+}}+H_{e}-H_{AH},$$

where $H_{{\left[AH\cdot \right]}^{+}}$ and $H_{e}$ represent the enthalpies of the radical cation and the electron, respectively. The proton transfer is described by the proton dissociation enthalpy (PDE),

(10) $$PDE=H_{A\cdot }+H_{H^{+}}-H_{{\left[AH\cdot \right]}^{+}},$$

where $H_{H^{+}}$ is the enthalpy of the proton. Intermolecular H-bonds between the antioxidant and the polar solvent hinder the H atom transfer process in the HAT mechanism. For this reason, the SET-PT mechanism is often favored in such environments [49].

The SPLET mechanism also occurs in two steps. In the first step, the antioxidant transfers a proton to the free radical, forming the anion [A**:**]^−^ and the cation [AH**∙**]^+^. In the second step, an electron is transferred from [A**:**]^−^ to [AH**∙**]^+^, forming A**⋅** and R–H. The thermodynamic descriptors associated with this mechanism are proton affinity

(11) $$PA=H_{{\left[A\cdot \right]}^{-}}+H_{H^{+}}-H_{AH},$$

and electron transfer enthalpy,

(12) $$ETE=H_{A\cdot }+H_{e}-H_{{\left[A\cdot \right]}^{-}},$$

where $H_{{\left[A\cdot \right]}^{-}}$ represents the enthalpy of the anion [A∶]^−^. The SPLET mechanism is solvent-dependent, with the first step mainly influenced by the solvent, as it involves the formation of electronically charged species [60].

### 3.3. Docking Analysis

After the evidence of the potential interaction between luteolin and CYP17A1, the target PDB ID: 3RUK (Protein Data Bank, https://www.rcsb.org/) was selected for docking analysis to construct interaction models [32]. Like gefitinib, luteolin causes growth arrest of PC-3 human prostate cancer cells, suggesting its potential against this kind of cancer and raising the necessity of possible associated mechanisms [61]. Many conformations of luteolin were simulated (10 best poses were finally selected) in complex with the structure of the biological target specified and the luteolin to verify the fit favorably into the ligand binding site of the biomolecule. The target protein was downloaded, and the water molecules were removed. After identifying the active site, the dimensions adopted for the docking were a grid box of 10 Å. The genetic algorithm was employed. The program GOLD Suite 5.7.0 (Mark Thompson and Planaria Software LLC) was used to generate the docking models. The 2D interaction maps were produced using Discovery Studio 3.5 Visualizer software (https://discover.3ds.com/discovery-studio-visualizer-download accessed on 18 January 2025), and the 3D images were built with PyMOL Molecular Graphics System, Version 3.1.3 software (https://www.pymol.org/). Redocking was performed using a structure in which the target protein and its ligand were co-crystallized to validate the models produced with our selected molecules. Default values were employed for all other parameters, and structures were submitted to 10 genetic algorithm runs using the CHEMPLP fitness function.

### 3.4. Generation of Shared Feature Pharmacophore Modeling of Ligands

To create shared features and pharmacophore models for selective ligands of the Cytochrome P450 17A1, we employed LigandScout v4.5 (https://docs.inteligand.com/ligandscout/ accessed on 9 February 2025) [37]. LigandScout identified combinations of shared pharmacophoric features, including hydrogen-bond donors (HBDs), hydrogen-bond acceptors (HBAs), positively and negatively charged groups, hydrophobic (HyPho) regions, and aromatic rings (ARs) [62]. These unique pharmacophores were subsequently integrated into the alignment process to develop the Shared Feature Pharmacophore (SFP) model. A literature review was conducted using the databases in Binding DB (https://www.bindingdb.org/rwd/bind/index.jsp accessed on 9 February 2025) to identify compounds with Cytochrome P450 17A1 antagonist activity. Five compounds with the lowest inhibitory concentration (IC_50_) values were included in a dataset used to obtain pharmacophore models.

## 4. Conclusions

The molecular modeling results indicate that the frontier molecular orbitals of luteolin are sensitive to solvent polarity, which may slightly influence its chemical reactivity. Furthermore, both experimental and theoretical studies in the literature confirm the significant antioxidant potential of luteolin, particularly in free radical scavenging mechanisms. The findings of this study suggest that intermolecular interactions between the OH groups of luteolin and water molecules subtly affect the dissociation energies of the O–H bonds during oxidative processes. Luteolin’s antioxidant activity is crucial in cellular protection against oxidative stress, which is a key factor in developing various diseases, including cancer and neurodegenerative disorders. By scavenging reactive oxygen species (ROS) and modulating essential enzymatic pathways, luteolin enhances cellular defense mechanisms, thereby promoting overall health. Furthermore, the association of pharmacophore modeling and molecular docking suggests the possibility that luteolin interferes with CYP17A1 as a potential mechanism against prostate cancer. Additionally, the analysis of its possible interactions with Cytochrome CYP17A1 suggests that luteolin may act as a modulator of enzymatic activity. Its pharmacophoric similarity to known ligands of CYP17A1 further reinforces its therapeutic potential. Catalytic processes involving CYP17A1 can generate free radicals as byproducts, contributing to cellular oxidative stress. The accumulation of these reactive species can lead to cellular damage, inflammation, and the onset of various pathologies, including cancer. In particular, CYP17A1 dysfunction and the subsequent increase in oxidative stress are closely linked to tumorigenesis, especially in hormone-dependent cancers such as prostate cancer. These findings underscore the importance of computational techniques, including DFT, pharmacophoric modeling, and molecular docking, in elucidating the mechanisms underlying the biological activity of luteolin. By deepening our understanding of its molecular interactions, this study opens new perspectives for developing therapeutic strategies targeting diseases associated with oxidative stress.

## Figures and Tables

**Table 1:** Geometric parameters and topological parameters of the QTAIMs of luteolin–solvent interactions obtained at the M06-2X/6-311++G(d,p) level of theory. The binding energy (BE) was calculated by Equation (1) from the ρ(r) values.

BCP	Interaction	Length (Å)	Angle (°)	ρ(r) (a.u.)	$\nabla^{2}\rho$ (a.u.)	G(r) (a.u.)	v(r) (a.u.)	h(r) (a.u.)	$\frac{\left|v\right|}{G}$	BE (kcal/mol)
Luteolin + Water										
I	$O_{1}\cdots$H–O*_w_*	1.92185	176.9067	0.0222	0.0952	0.0208	−0.0177	0.0030	0.9	−8.43
II	$O_{2}–H\cdots$O*_w_*	1.63838	171.2233	0.0308	0.1254	0.0296	−0.0279	0.0017	0.9	−11.29
III	$O_{2}\cdots$H–O*_w_*	1.88308	165.6301	0.0230	0.0977	0.0215	−0.0186	0.0029	0.9	−8.71
VI	$O_{3}–H\cdots$O*_w_*	1.76759	163.1268	0.0389	0.1314	0.0346	−0.0363	−0.0017	1.0	−14.00
V	$O_{3}\cdots$H–O*_w_*	2.15803	129.3470	0.0207	0.0852	0.0188	−0.0162	0.0026	0.9	−7.95
VI	$O_{4}–H\cdots$O*_w_*	1.68388	170.2508	0.0308	0.1210	0.0289	−0.0275	0.0014	1.0	−11.31
VII	$O_{4}\cdots$H–O*_w_*	1.97618	156.8234	0.0227	0.0969	0.0216	−0.0190	0.0026	0.9	−8.61
Luteolin + DMSO										
I	$O_{1}\cdots$H–O*_d_*	1.81451	140.9443	0.0313	0.1232	0.0298	−0.0287	0.0011	1.0	−11.45
II	$O_{2}\cdots$H–O*_d_*	1.67271	161.178	0.0478	0.1499	0.0429	−0.0484	−0.0055	1.1	−16.94
III	$O_{3}\cdots$H–O*_d_*	1.74762	167.7532	0.0385	0.1403	0.0363	−0.0375	−0.0012	1.0	−13.85
IV	$O_{4}\cdots$H–O*_d_*	1.75890	155.9998	0.0396	0.1374	0.0363	−0.0383	−0.0020	1.1	−14.22
Luteolin + Methanol										
I	$O_{1}\cdots$H–O*_m_*	2.02554	143.9005	0.0206	0.0838	0.0187	−0.0164	0.0023	0.9	−7.92
II	$O_{2}–H\cdots$O*_m_*	1.76984	160.7465	0.0352	0.1358	0.0336	−0.0333	0.0003	1.0	−12.76
III	$O_{2}\cdots$H–O*_m_*	1.98083	149.8292	0.0228	0.0920	0.0208	−0.0186	0.0022	0.9	−8.63
IV	$O_{3}–H\cdots$O*_m_*	1.74293	150.5949	0.0393	0.1349	0.0355	−0.0372	−0.0018	1.0	−14.13
V	$O_{3}\cdots$H–O*_m_*	1.99651	142.3781	0.0210	0.0868	0.0191	−0.0166	0.0026	0.9	−8.03
VI	$O_{4}–H\cdots$O*_m_*	1.77792	163.0294	0.0345	0.1335	0.0329	−0.0324	0.0005	1.0	−12.54
VII	$O_{4}\cdots$H–O*_m_*	2.01683	133.3419	0.0221	0.0904	0.0204	−0.0183	0.0021	0.9	−8.42
Radical + Water										
I	O_1_...H–O*_w_*	1.89589	150.992	0.0249	0.1082	0.0241	−0.0211	0.0029	0.9	−9.34
II	O_1_...H–O*_w_*	2.33842	121.682	0.0121	0.0457	0.0102	−0.0090	0.0012	0.9	−5.08
III	O_2_...H–O*_w_*	1.77020	174.843	0.0240	0.0970	0.0218	−0.0193	0.0025	0.9	−9.04
IV	O_2_...H–O*_w_*	1.66756	172.960	0.0275	0.1112	0.0255	−0.0233	0.0023	0.9	−10.22
V	O_3_...H–O*_w_*	1.96877	155.187	0.0217	0.0871	0.0193	−0.0168	0.0025	0.9	−8.28
VI	O_4_...H–O*_w_*	2.23303	118.390	0.0152	0.0598	0.0133	−0.0116	0.0016	0.9	−6.10
VII	O_4_...H–O*_w_*	1.82504	177.968	0.0312	0.1184	0.0284	−0.0272	0.0012	1.0	−11.43

**Table 2:** Second-order perturbation theory analysis in the NBO basis obtained at the M06-2X/6-311++G(d,p) level of theory for the interactions of the OH groups of luteolin with solvent molecules.

Interaction	Hyperconjugation	$E_{i\to j^{*}}^{\left(2\right)}$ (kcal/mol)	Donor		Acceptor	
			Occupancy	Hybrid	Occupancy	Hybrid
Luteolin + Water						
$O_{1}\cdots$H–O*_w_*	$\eta_{1}$$(O_{1})\;\to$ $\sigma^{*}$(O*_w_*–H)	5.09	1.96714	O_1_: *sp*^1.52^	0.01157	O*_w_*: *sp*^2.85^ H: *s*
$O_{2}\cdots$H–O*_w_*	$\eta_{1}$$(O_{2})\;\to$ $\sigma^{*}$(O*_w_*–H)	5.87	1.96837	O_2_: *sp*^1.57^	0.01279	O*_w_*: *sp*^2.82^ H: *s*
$O_{2}–H\cdots$O*_w_*	$\eta_{2}$$(O_{w})\;\to$ $\sigma^{*}$(O_2_–H)	13.81	1.97236	O*_w_*: *sp*^1.06^	0.02861	O_2_: *sp*^2.97^ H: *s*
$O_{3}\cdots$H–O*_w_*	$\eta_{2}$$(O_{3})\;\to$ $\sigma^{*}$(O*_w_*–H)	2.56	1.87545	O_3_: *p*	0.01088	O*_w_*: *sp*^2.86^ H: *s*
$O_{3}–H\cdots$O*_w_*	$\eta_{2}$$(O_{w})\;\to$ $\sigma^{*}$(O_3_–H)	21.0	1.95287	O*_w_*: *sp*^4.56^	0.04652	O_3_: *sp*^2.83^ H: *s*
$O_{4}\cdots$H–O*_w_*	$\eta_{2}$$(O_{4})\;\to$ $\sigma^{*}$(O*_w_*–H)	3.46	1.97141	O_4_: *sp*^1.63^	0.00902	O*_w_*: *sp*^2.87^ H: *s*
$O_{4}–H\cdots$O*_w_*	$\eta_{2}$$(O_{w})\;\to$ $\sigma^{*}$(O_4_–H)	13.46	1.97099	O*_w_*: *sp*^1.69^	0.02908	O_4_: *sp*^3.10^ H: *s*
Luteolin + DMSO						
$O_{1}–H\cdots$O*_d_*	$\eta_{1}$$(O_{d})\;\to$ $\sigma^{*}$(O_1_–H)	4.49	1.98584	O*_d_*: *sp*^0.30^	0.04233	O_1_: *sp*^2.66^ H: *s*
$O_{2}–H\cdots$O*_d_*	$\eta_{2}$$(O_{d})\;\to$ $\sigma^{*}$(O_2_–H)	20.57	1.89740	O*_d_*: *p*	0.05544	O_2_: *sp*^2.89^ H: *s*
$O_{3}–H\cdots$O*_d_*	$\eta_{3}$$(O_{d})\;\to$ $\sigma^{*}$(O_3_–H)	12.72	1.86769	O*_d_*: *p*	0.03811	O_3_: *sp*^2.86^ H: *s*
$O_{4}–H\cdots$O*_d_*	$\eta_{2}$$(O_{d})\;\to$ $\sigma^{*}$(O_4_–H)	14.74	1.91064	O*_d_*: *p*	0.04176	O_4_: *sp*^2.90^ H: *s*
Luteolin + Methanol						
$O_{1}\cdots$H–O*_m_*	$\eta_{1}$$(O_{1})\;\to$ $\sigma^{*}$(O*_m_*–H)	3.59	1.96886	O_1_: *sp*^1.52^	0.01371	O*_m_*: *sp*^2.85^ H: *s*
$O_{2}\cdots$H–O*_m_*	$\eta_{1}$$(O_{2})\;\to$ $\sigma^{*}$(O*_m_*–H)	4.43	1.96914	O_2_: *sp*^2.19^	0.01613	O*_m_*: *sp*^3.39^ H: *s*
$O_{2}–H\cdots$O*_m_*	$\eta_{2}$$(O_{m})\;\to$ $\sigma^{*}$(O_2_–H)	13.70	1.95560	O*_m_*: *sp*^2.84^	0.03314	O_2_: *sp*^2.96^ H: *s*
$O_{3}\cdots$H–O*_m_*	$\eta_{2}$$(O_{3})\;\to$ $\sigma^{*}$(O*_m_*–H)	2.07	1.87565	O_3_: *p*	0.01572	O*_m_*: *sp*^3.35^ H: *s*
$O_{3}–H\cdots$O*_m_*	$\eta_{2}$$(O_{m})\;\to$ $\sigma^{*}$(O_3_–H)	18.63	1.93513	O*_m_*: *sp*^6.17^	0.04631	O_3_: *sp*^2.83^ H: *s*
$O_{4}\cdots$H–O*_m_*	$\eta_{2}$$(O_{4})\;\to$ $\sigma^{*}$(O*_m_*–H)	3.22	1.97194	O_4_: *sp*^1.56^	0.01320	O*_m_*: *sp*^3.42^ H: *s*
$O_{4}–H\cdots$O*_m_*	$\eta_{2}$$(O_{m})\;\to$ $\sigma^{*}$(O_4_–H)	13.40	1.95535	O*_m_*: *sp*^3.15^	0.03252	O_4_: *sp*^2.97^ H: *s*
Radical + Water						
[O_1_**∙**]⋯H–O*_w_*	$\eta_{2}$(O_1_**∙**) → $\sigma^{*}$(O*_w_*–H)	0.31	0.95732	O_1_**∙**: *p*	0.00906	O*_w_*: *sp*^2.72^ H: *s*
[O_2_**∙**]⋯H–O*_w_*	$\eta_{1}$(O_2_**∙**) → $\sigma^{*}$(O*_w_*–H)	1.18	0.98396	O_2_**∙**: *sp*^0.69^	0.01036	O*_w_*: *sp*^2.67^ H: *s*
[O_2_**∙**]⋯H–O*_w_*	$\eta_{2}$(O_2_**∙**) → $\sigma^{*}$(O*_w_*–H)	3.35	0.95440	O_2_**∙**: *p*	0.01071	O*_w_*: *sp*^2.66^ H: *s*
[O_3_**∙**]⋯H–O*_w_*	$\eta_{3}$(O_3_**∙**) → $\sigma^{*}$(O*_w_*–H)	1.64	0.88284	O_3_**∙**: *p*	0.00857	O*_w_*: *sp*^2.76^ H: *s*
[O_4_**∙**]⋯H–O*_w_*	$\eta_{2}$(O_4_**∙**) → $\sigma^{*}$(O*_w_*–H)	5.12	0.95218	O_4_**∙**: *p*	0.01359	O*_w_*: *sp*^2.58^ H: *s*

**Table 3:** Bond dissociation enthalpy (BDE), ionization potential (IE), proton dissociation enthalpy (PDE), proton affinity (PA), and electron transfer enthalpy (ETE) of radicals of the luteolin formed during the free radical scavenging processes. These values were calculated using the M06-2X/6-311++G(d,p) level of theory.

Compound	BDE (kcal/mol)		IP + PDE (kcal/mol)	PA + ETE (kcal/mol)
Luteolin				
	No Water	Water		
O_1_–H	88.072	88.681	91.133	91.061
O_2_–H	92.153	93.544	95.213	95.142
O_3_–H	80.662	82.757	83.722	83.651
O_4_–H	83.605	82.096	86.665	86.594
Butylhydroxyanisole (BHA)				
O_1_–H	77.161		80.213	80.150
Butylhydroxytoluene (BHT)				
O_1_–H	76.690		85.515	79.679
Gallic acid (GA)				
O_1_–H	84.056		87.118	87.045
O_2_–H	81.193		84.255	84.182
Propyl gallate (PG)				
O_1_–H	82.836		85.897	85.825
O_2_–H	78.963		82.025	81.952
Pyrogallol (PY)				
O_1_–H	81.331		84.388	84.320
O_2_–H	78.040		81.098	81.029
*Tert*-butylhydroquinone (TBHQ)				
O_1_–H	77.459		91.776	80.447
O_2_–H	78.548		92.866	81.537

## Data Availability

Data is contained within the article.

## Abbreviations

The following abbreviations are used in this manuscript:

DFT	Density Functional Theory
HOMO	Highest Occupied Molecular Orbital
LUMO	Lowest Unoccupied Molecular Orbital
MEP	Molecular Electrostatic Potential
QTAIMs	Quantum Theory of Atoms in Molecules
NBO	Natural Bond Orbital
